# Efficacy of a proprietary combination of *Tamarindus indica* seeds and *Curcuma longa* rhizome extracts in osteoarthritis: a clinical investigation

**DOI:** 10.29219/fnr.v67.9268

**Published:** 2023-06-20

**Authors:** Nandlal Prasad, Vineet Vinay, Anupam Srivastava

**Affiliations:** 1 Bajrang Memorial Fracture, Accidental & Surgical Center, Varanasi, Uttar Pradesh, India; 2 The p value (RES & STATS Private Limited), Pune, Maharashtra, India; 3 Vatsalya Multispeciality Hospital, Varanasi, Uttar Pradesh, India

**Keywords:** joint pain, musculoskeletal functions, NXT15906F6, osteoarthritis, safety, WOMAC

## Abstract

**Background:**

NXT15906F6 (TamaFlex™) is a proprietary blend containing standardized *Tamarindus indica* seeds and *Curcuma longa* rhizome extracts. Earlier, NXT15906F6 supplementation demonstrated reduced knee joint pain and improved musculoskeletal functions in healthy and knee osteoarthritis (KOA) subjects.

**Objective:**

The present randomized, double-blind, placebo-controlled study was focused on validating the clinical efficacy of NXT15906F6 in a larger number of subjects with KOA.

**Methods:**

Male and female subjects (age: 40–70 years; body mass index [BMI]: 20–29 kg/m^2^] were randomized into three groups receiving placebo (*n* = 50), NXT15906F6 (*n* = 50) or a blend of *C. longa* and *Boswellia serrata* extracts (CLBS) (*n* = 50). Subjects consumed 250 mg NXT15906F6, 1,000 mg CLBS or a matched placebo daily after breakfast over a period of 30 consecutive days. The primary efficacy outcome was the improvement in total Western Ontario and McMaster Universities Osteoarthritis Index (WOMAC) scores, and the secondary efficacy measures included various tests on joint pain and musculoskeletal functions and evaluations ofserum high-sensitivity C-reactive protein (hs-CRP) and a cartilage degradation marker, C-terminal telopeptide of type II collagen in urine (uCTX-II).

**Results:**

NXT15906F6 significantly (*P* < 0.001) reduced the WOMAC scores and improved musculoskeletal function scores in the participants as compared with baseline and placebo. NXT15906F6 participants reduced knee pain and improved musculoskeletal functions as early as day 5 of supplementation. In contrast, CLBS supplementation failed to show early efficacies in pain and functional scores, except for 30s-CST and primary knee flexion. The NXT1506F6-supplemented participants significantly reduced serum hs-CRP and uCTX-II levels from baseline and as compared with the placebo. Both supplementations did not alter the subjects’ clinical chemistry, hematology, and vital parameters.

**Conclusion:**

The anti-inflammatory botanical composition NXT15906F6 supplementation mitigated joint pain and improved musculoskeletal functions and joint motility in KOA subjects. This botanical composition was also well-tolerated by the volunteers.

## Popular scientific summary

- NXT15906F6 (TamaFlex™) is a food-derived, synergistic anti-inflammatory composition of *T. indica* seeds and *C. longa* rhizome extracts.
- The present 30-day trial validates the anti-OA efficacy of NXT15906F6 supplementation in mitigating knee joint pain and improving musculoskeletal functions and joint motility in OA subjects.
- Significant clinical benefit starts as early as day 5 of NXT15906F6 supplementation.
- NXT15906F6 is well-tolerated by the volunteers.

OA has been an underlying cause of joint pain and disability, and approximately 500 million people globally suffer from it, causing a significant socioeconomic impact on health care (1). Commonly, knee OA affects older adults, who account for 12% of the aging population suffering from continuing knee pain, and has been estimated as the fourth vital cause of disability (2). The World Health Organization showed that globally 10–15% of adults currently have arthritis and a two-fold increase is anticipated by 2030 (3, 4). According to an estimate by the Centers for Disease Control and Prevention (CDC), one in four US adults has some form of arthritis, and it is projected to reach 78 million by the year 2040 (5).

The most commonly used drugs in the management of OA include non-steroidal anti-inflammatory drugs (NSAIDs), intra-articular hyaluronic acid, or corticosteroids (6). However, their long-term use induces adverse effects. Therefore, medicinal plants have been explored adequately to overcome such adverse events (7). Previous studies have shown that the synergistic activity of curcumin and boswellic acids reduced the dosage and intake frequency of acetaminophen and NSAIDs (8, 9). Despite advanced treatment options, no substantial therapy can curb the disease progression or avert structural damage (10). Therefore, there is a growing need to develop safe and efficacious plant-based formulations that can substantially mitigate OA pathology without exerting toxic manifestations.

NXT15906F6 (TamaFlex^TM^) is a blend of aqueous and ethanolic extracts of *T. indica* (tamarind) seeds and ethanolic extract of *C. longa* rhizome. NXT15906F6 comprises a minimum of 65% of proanthocyanidins and 3% of total curcuminoids (11). Earlier clinical studies demonstrated the substantial protective efficacy of NXT15906F6 against joint pain and inflammation (11, 12).

The use of tamarind seeds is on the rise primarily due to their proven therapeutic efficacy against diabetes, diarrhea, dysentery, and ulcers (7). Tamarind seed extracts (TSE) are enriched in triterpenes, procyanidins, polyphenolic bioactive compounds, and polysaccharides, which exert significant protective and therapeutic efficacies against various human pathophysiological ailments (13). Tamarind seed kernel offers potent antioxidant and anti-inflammatory activities. The anti-arthritic efficacy of TSE was studied in arthritis-induced rats (14). Curcumin has been used in the treatment of manifold diseases for ages due to its robust anti-inflammatory and anti-oxidative effects (15). Accordingly, a few clinical studies have shown that oral administration of curcumin mitigates pain and improves physical function (16, 17). *In vivo* studies have demonstrated that curcumin attenuates OA progression by reducing inflammation by hindering the Toll-like receptor/nuclear factor-κβ (TLR4/NF-κβ) signaling pathway and suppressing chondrocyte apoptosis through augmented apoptosis (18).

The present follow-up compliance clinical investigation was undertaken on a larger number of participants with OA in a different geographical location in India to validate the clinical efficacy of NXT15906F6. This study was conducted in northern India, while the earlier study was performed in the southern part of India (11). Also, this study was intended to evaluate the efficacy of NXT15906F6 in parallel with a clinically proven and a widely investigated anti-inflammatory botanical blend of curcuminoids and boswellic acids that reported to mitigate joint pain and improve musculoskeletal functions in OA subjects (9). This 30-day randomized, placebo-controlled trial evaluated the primary clinical symptoms of OA, such as joint pain, stiffness, and physical function in the volunteers, improvements in musculoskeletal functions, an inflammatory marker in serum, and a cartilage degradation marker in the urine samples of the participants. The study’s secondary objective was to assess the tolerability of the botanical formulation.

## Materials and methods

### Study material

NXT15906F6 (TamaFlex^TM^) is an herbal formulation prepared using *T. indica* seeds and *C. longa* rhizome extracts. The powdered dry *T. indica* seeds were extracted with aqueous ethanol and followed by water. These extracts were concentrated separately and blended in a 9:1 ratio to obtain the *T. indica* seed extract. The powdered dry *C. longa* rhizomes were extracted with aqueous ethanol to obtain *C. longa* rhizome extract. Each extract was concentrated under a vacuum at room temperature. NXT15906F6 comprises *T. indica* seed extract, *C. longa* rhizome extract and excipients in 6:3:1 ratio (w/w). The excipient was a mixture of microcrystalline cellulose powder and Syloid silica in an 8:2 ratio (w/w). NXT15906F6 was standardized to comprise at least 65% of proanthocyanidins and 3% of total curcuminoids (11).

CLBS is a blend containing standardized extracts of *C. longa* rhizome and *B. serrata* gum resin. Each 500 mg of CLBS consists of 333.3 mg *C. longa extract* containing approximately 95% total curcuminoids and 166.7 mg of *B. serrata* extract standardized to 80% total boswellic acids.

### Clinical study design

The present randomized, double-blind, placebo-controlled clinical investigation determined the efficacy and tolerability of NXT15906F6 in mild to moderate osteoarthritic adult subjects. This study was conducted at two sites (Vatsalya Hospital Multispeciality Center and Bajarang Memorial Fracture, Accidental & Surgical Center) in Varanasi, Uttar Pradesh, India, according to the ICH-GCP guidelines. The study protocol and related documents were reviewed and approved by the Institutional ethics committee (Vatsalya Ethics Committee). The study protocol was registered in the Clinical Trial Registry of India (CTRI/2021/12/039022). This trial was conducted between Dec. 2021 and May 2022. Among 150 participants, 44, 46, and 45 subjects in the placebo, NXT15906F6, and the CLBS groups completed the study. All participants signed written informed consent.

Male and female participants were between 40 and 70 years of age with a body mass index (BMI) between 20 and 29 kg/m^2^. They had either unilateral or bilateral OA of the knee according to the criteria of the American College of Rheumatology-Kellgren-Lawrence (KL) grade II based on X-ray examination (19) and visual analog scale (VAS) score between 40 and 70 mm (20). Primarily, the subjects were screened through the inclusion–exclusion criteria (Table 1) and recruited into the intervention.

**Table 1:** Inclusion and exclusion criteria

Inclusion criteria	Exclusion criteria
Ambulatory, healthy male and female subjects (age: 40–70 years) with Body Mass Index (BMI) between 20 and 29 kg/m^2^	Previous injury and/or surgery to the knee. History of underlying inflammatory arthropathy, rheumatoid arthritis (RA) or Systemic Lupus Erythematosis (SLE)
Unilateral or bilateral knee osteoarthritis grade II of Kellgren and Lawrence based on X-ray examination	Uncontrolled diabetes (FPG>125 mg/dL) and Hypertension (Systolic > 120 mmHg and Diastolic >80 mmHg). Subjects suffering from COPD or having history of any respiratory or breathing disorders
Visual analog scale (VAS) score between 40 and 70 mm	A history of immune system and autoimmune disorders or use of immunosuppressive drugs in past 6 months
Routine hematology, clinical biochemistry parameters and vital signs within the normal ranges	Hypersensitivity to NSAIDs, abnormal liver or kidney function tests, history of peptic ulceration and upper GI hemorrhage, congestive heart failure, hypertension, hyperkalemia.
Availability during the study period and refrain from using any natural health products including, glucosamine and chondroitin one month prior to and during the study.	Pregnant, breastfeeding or planning to become pregnant during the study.
	Consumption of acetaminophen / paracetamol, ibuprofen, aspirin or other NSAIDS or any other pain reliever (OTC or prescription) or any herbal products within 7 days prior to the screening visit
-	High alcohol intake (>2 standard drinks per day) or use of psychotropic drugs
-	History of cardiovascular, lung cancer or chronic diseases. Subjects with HIV positive

**Fig. 1:**
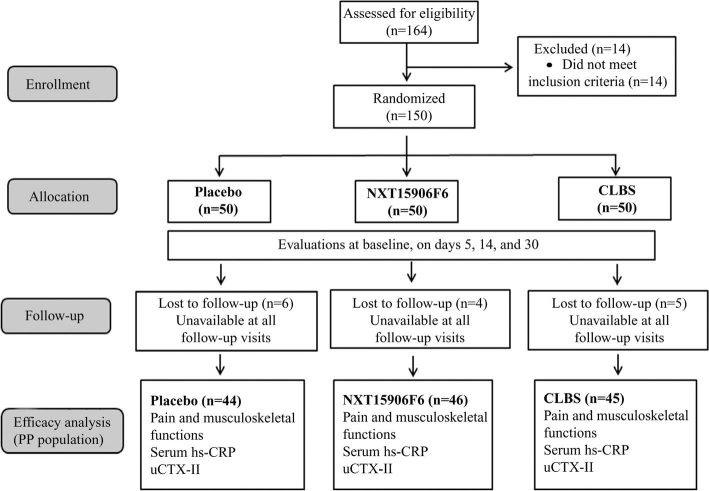
CONSORT diagram represents the recruitment of the study participants and flow of the trial process. Knee pain and musculoskeletal functions were assessed at baseline and on days 5, 14, and 30 of the study. Serum hs-CRP and urinary C-terminal cross-linked telopeptide of type II collagen (uCTX-II) were measured at baseline and end of the study.

Participants were randomly assigned to one of the three groups (*n* = 50), placebo, NXT15906F6-250 mg, and CLBS-1,000 mg. The participants were allocated into groups using computer-generated block randomization utilizing the SAS PROC-PLAN (SAS Institute Inc.), as described earlier (11). The investigational products (IP) were encapsulated in similar appearance and size capsules packed in identical opaque high-density polyethylene (HDPE) bottles. The IP bottles were sequentially numbered as per the randomization code and the enrolled participants received the IP bottles according to their respective randomization numbers. Participants received two identical capsules containing a placebo, NXT15906F6, or CLBS daily after breakfast. The study had five visits, visit 1 (screening visit), visit 2 or baseline (randomization visit), visit 3 (first follow-up at day 5), visit 4 (second follow-up at day 14), and visit 5 (third follow-up at day 30). The subject allocation into three groups and the intervention flow are summarized in a CONSORT flow diagram (Fig. 1).

### Primary outcome measure

WOMAC score measurement is the primary efficacy measure of this clinical study compared with the placebo. WOMAC score in the range of 0 to 100 describes joint pain, stiffness, and physical function in subjects with knee OA (21). The total index comprises 24 questions; WOMAC A (pain) subscale contains five questions; WOMAC B (stiffness) and WOMAC C (physical functioning) subscales include 19 questions (2 stiffness and 17 physical functioning questions). WOMAC scores were evaluated at baseline and on days 5, 14, and 30 of the study.

### Secondary outcome measures

The secondary efficacy evaluations are improvement in the scores of 1) VAS, 2) Lequesne’s functional index (LFI), 3) the Six Minute Walk Test (SMWT), 4) Stair Climb Test (SCT), 5) knee flexion range of motion (ROM), and 6) 30-sec chair stand test (30s-CST) at the end of the study from baseline. VAS, LFI, SMWT, SCT, and ROM of the primary knee were measured following the standard methods described earlier (12). The 30s-CST is a reliable and valid test for assessing lower body strength and functional performance in aging and older adults. This test was performed as previously described (22). These pain and musculoskeletal functional evaluations were carried out at baseline and on days 5, 14, and 30 of supplementation. In addition, this study also evaluated biomarkers such as high-sensitivity C-reactive protein (hs-CRP) in serum samples and C-terminal cross-linked telopeptide of type II collagen (CTX-II) in the urine samples of the volunteers at baseline and on day 30.

### Serum and urine biomarkers

The study participants’ serum hs-CRP and urinary CTX-II (uCTX-II) were evaluated using commercial ELISA kits, following the manufacturer’s instructions. The hs-CRP (Cat# E-EL-H5134) ELISA kits were procured from Elabscience (Houston, TX); each serum sample was run in duplicate wells of the test plates. The pre-coated 96-well assay plates were incubated with the serum samples, the bound analyte was probed with the biotinylated detection antibody, and the signal was detected by an enzyme-chromogen detection method as specified. The developed color reaction was measured in a microplate reader (Bio-Rad Laboratories, Hercules, CA). The minimum detection limits of hs-CRP and CTX-II assays are 9.38 pg/mL and 0.10 ng/mL, respectively.

The measured CTX-II concentrations were normalized to the creatinine concentrations in the respective urine samples. Urinary creatinine was measured using creatinine assay reagents (Cat# 0018255540; Instrumentation Laboratory, Milan, Italy) following the instructions provided by the manufacturer. The assay method was based on the color reaction of creatinine with picric acid under an alkaline condition. The formation of a red-colored complex was proportional to the quantity of creatinine in the urine sample. The absorbance was measured at 510 nm in a pre-calibrated, automated biochemistry analyzer (ILAB Aries, Instrumentation Laboratory, Monza, Italy). The normalized uCTX-II was expressed in ng/mmol creatinine (Cr).

### Safety parameters

Safety evaluations were carried out using an array of hematological, serum, biochemical measurements, and urinalysis at the screening and end of the study. Serum biochemical and hematological parameters were analyzed using an automated analyzer (Siemens Dimension Xpand Plus, NY, USA) and a hematological counter (Coulter LH-750, Beckman Coulter Inc., IN, USA). Urinalysis was done using a urine analysis kit (Roche Diagnostics, IN, USA). Microscopic examinations were conducted under a clinical light microscope (Olympus Opto Systems India Pvt. Ltd., New Delhi, India). In addition, this study also evaluated the participants’ vital signs at every visit of the study. The vital signs were blood pressure (systolic/diastolic), pulse rate, respiratory rate, and body temperature.

Adverse events monitoring was strictly enforced in the volunteers throughout the intervention.

### Rescue medication

Paracetamol (2,000 mg/day) was recommended as rescue medication based on the investigator’s advice. Accordingly, its use was recorded in the source documents and case report form.

### Statistical analysis

Forty-five subjects per arm were chosen by assuming the effect size (d) 2.5 (90% power at 95% CI) with a standard deviation (d) of 2.6 in analgesic efficacy of NXT15906F6 on WOMAC Pain scores. Data from 135 subjects, who completed the study with a minimum of 80% compliance, presented (mean ± SD) as per protocol (PP) population. Analysis of covariance (ANCOVA) and paired *t*-tests were used to analyze the mean differences ‘between the groups’ and ‘within the group’, respectively. Safety analysis was performed on the participants who received at least one dose of the study supplements. Paired *t*-test was used to evaluate baseline characteristics, vital signs, and laboratory parameters. The analyses were performed using SPSS version 2.2, and a *P* < 0.05 was considered statistically significant.

## Results

### Study participants

A total of 135 participants completed this 30-day study. Participants had knee osteoarthritis KL grade II based on X-ray examination (19). The subjects’ baseline characteristics were not statistically different in ‘between-the-groups’. Table 2 summarizes participants’ demographics and baseline characteristics (ITT population).

**Table 2:** Demography and baseline characteristics

Parameters	Placebo (*n* = 50)	NXT15906F6-250 mg (*n* = 50)	CLBS-1000 mg (*n* = 50)	*P*
Age, years	53.4 ± 9.26 (40–70)	53.1 ± 9.09 (40–70)	51.04 ± 8.06 (40–67)	0.8705^a^, 0.1771^b^
Gender, M + F	28 + 22	30 + 20	26 + 24	-
Body weight, kg	66.24 ± 7.24 (52.5–82)	66.16 ± 6.86 (51.5–80.5)	65.41 ± 7.69 (49.5–83.5)	0.9549^a^, 0.5798^b^
Height, cm	160.6 ± 6.10 (150–170)	161.78 ± 5.71 (149–170)	160.44 ± 6.57 (148–172)	0.3203^a^, 0.8998^b^
BMI, kg/m^2^	25.61 ± 1.31 (23–28.4)	25.21 ± 1.40 (22.2–28.5)	25.32 ± 1.53 (21.4–28.5)	0.1484^a^, 0.3252^b^

Data presented as mean ± SD. ^a^ and^b^ indicate intergroup analyses placebo versus NXT15906F6, and placebo versus CLBS group, respectively, using *t*-test, unequal variance. Data ranges are indicated in parentheses.

Six, four, and five subjects in the placebo, NXT15906F6, and CLBS groups were lost to follow-up; they were unavailable during the follow-up visits. So, 135 participants completed the study; placebo (*n* = 44), NXT15906F6 (*n* = 46), and CLBS group (*n* = 45).

### Efficacy of NXT15906F6

#### Total WOMAC score

**Fig. 2:**
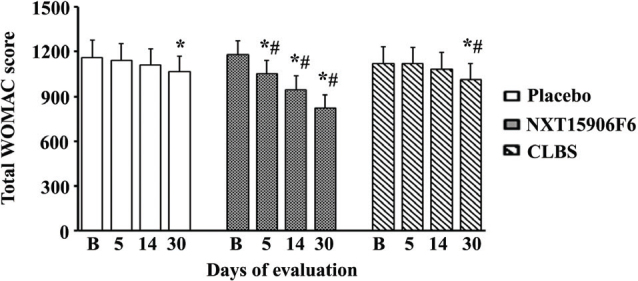
Total WOMAC scores in the participants. Data represent mean ± SD in Placebo (*n* = 44), NXT15906F6 (*n* = 46), and CLBS (*n* = 45) supplemented subjects at baseline (B) and on days 5, 14, and 30 of the study. * and ^#^ indicate significance (*P* < 0.05) in comparison analyses versus baseline (B) and versus placebo, respectively.

Participants supplemented with NXT15906F6 showed gradual improvement in the total WOMAC score. The NXT15906F6 group showed significant reductions in the total WOMAC scores from day 5 through the end of the study. These reductions were significant (*P* < 0.001) as compared with the baseline and placebo. In the NXT15906F6 group, the reductions (*P* < 0.001) in total WOMAC scores were 10.84, 19.77, and 30.19% on days 5, 14, and 30, respectively, of supplementation as compared with baseline. In contrast, the CLBS group showed a significant reduction only on day 30 (9.97%, *P* < 0.001 vs. baseline). This change is also significant (*P* = 0.031) as compared with the placebo (Fig. 2).

#### WOMAC pain

From baseline, the NXT15906F6 group showed significant reductions (*P* < 0.001) in WOMAC pain scores by 19.79, 31.86, and 38.11% on days 5, 14, and 30, respectively. Post-trial, the placebo, and CLBS-supplemented groups also showed 15.12% (*P* < 0.001) and 17.43% (*P* < 0.001) and reductions in WOMAC-A subscale (Pain) scores as compared with baseline (Table 3).

**Table 3:** WOMAC Subscale scores

Measures	Groups	Evaluations	Score	Change from baseline	*P* (vs. baseline)	95% CI vs. baseline	*P* (vs. placebo)	95% CI vs. placebo
WOMAC- Pain	Placebo	Baseline	50.41 ± 6.1	-	-	-	-	-
		Day 5	48.82 ± 5.46	−1.59 ± 2.75	0.578	−0.86, 4.04	-	-
		Day 14	45.16 ± 6.12	−5.25 ± 4.38	<0.001	2.66, 7.83	-	-
		Day 30	42.79 ± 5.64	−7.62 ± 3.63	<0.001	5.13, 10.10	-	-
	NXT15906F6-250 mg	Baseline	48.65 ± 2.55	-	-	-	-	−0.18, 3.70
		Day 5	39.02 ± 3.54	−9.63 ± 2.95	<0.001	8.35, 10.90	<0.001	7.88, 11.72
		Day 14	33.15 ± 3.73	−15.50 ± 3.45	<0.001	14.17, 16.82	<0.001	9.89, 14.12
		Day 30	30.11 ± 3.85	−18.54 ± 3.72	<0.001	17.18, 19.89	<0.001	10.66, 14.70
	CLBS-1000 mg	Baseline	49.35 ± 5.87	-	-	-	-	−1.46, 3.59
		Day 5	47.75 ± 6.2	−1.60 ± 4.17	0.581	−0.92, 4.12	0.598	−1.39, 3.53
		Day 14	44.64 ± 5.77	−4.71± 4.57	<0.001	2.27, 7.14	0.891	−1.98, 3.02
		Day 30	40.75 ± 5.94	−8.60 ± 4.46	<0.001	6.12, 11.07	0.159	−0.40, 4.48
WOMAC- Stiffness	Placebo	Baseline	41.76 ± 5.1	-	-	-	-	-
		Day 5	42.78 ± 6.69	1.02 ± 4.39	0.829	−1.50, 3.54	-	-
		Day 14	40.23 ± 5.25	−1.53 ± 4.25	0.578	−0.66, 3.72	-	-
		Day 30	38.69 ± 5.32	−3.07 ± 4.27	0.055	0.86, 5.27	-	-
	NXT15906F6-250 mg	Baseline	41.41 ± 5.34	-	-	-	-	−1.84, 2.54
		Day 5	35.43 ± 5.28	−5.98 ± 2.20	0.001	3.78, 8.17	<0.001	4.83, 9.87
		Day 14	33.48 ± 5.07	−7.93 ± 2.95	<0.001	5.77, 10.08	<0.001	4.59, 8.91
		Day 30	28.91 ± 5.26	−12.50 ± 4.05	<0.001	10.30, 14.69	<0.001	7.56, 12.00
	CLBS-1000 mg	Baseline	40.61 ± 9.89	-	-	-	-	−2.18, 4.48
		Day 5	39.28 ± 8.54	−1.33 ± 5.05	0.892	−2.54, 5.21	0.049	0.26, 6.73
		Day 14	38.44 ± 8.3	−2.17 ± 5.90	0.616	−1.65, 5.99	0.387	−1.14, 4.72
		Day 30	36.17 ± 8.47	−4.44 ± 5.69	0.062	0.58, 8.29	0.165	−0.47, 5.51
WOMAC- Function	Placebo	Baseline	48.55 ± 5.46	-	-	-	-	-
		Day 5	47.69 ± 5.06	−0.86 ± 1.53	0.862	−1.37, 3.09	−	-
		Day 14	47.31 ± 5.04	−1.24 ± 2.34	0.671	−0.98, 3.46	-	-
		Day 30	45.58 ± 4.97	−2.97 ± 2.40	0.037	0.75, 5.18	-	-
	NXT15906F6-250 mg	Baseline	50.13 ± 5.38	-	-	-	-	−0.69, 3.85
		Day 5	46.15 ± 4.69	−3.98 ± 2.40	<0.001	1.88, 6.07	0.318	−0.50, 3.58
		Day 14	41.92 ± 5.1	−8.21 ± 3.50	<0.001	6.03, 10.38	<0.001	3.27, 7.52
		Day 30	36.12 ± 5.02	−14.00 ± 4.25	<0.001	11.85, 16.16	<0.001	7.37, 11.55
	CLBS-1000 mg	Baseline	46.78 ± 5.31	-	-	-	-	−0.50, 4.04
		Day 5	47.21 ± 5.37	0.44 ± 1.09	0.980	−1.80, 2.66	0.893	−1.72, 2.68
		Day 14	45.94 ± 5.53	−0.83 ± 1.24	0.884	−1.43, 3.11	0.434	−0.86, 3.60
		Day 30	43.24 ± 5.28	−3.54 ± 3.08	0.016	1.32, 5.75	0.079	0.18, 4.50

Data presented as mean ± SD. Placebo (*n* = 44), NXT15906F6 (*n* = 46) and CLBS (*n* = 45). ‘Within the group’ and ‘between the groups’ comparisons were analyzed using paired *t*-test and ANCOVA, respectively. *P* < 0.05 indicate significance.

#### WOMAC stiffness

NXT15906F6 supplementation significantly reduced the stiffness scores starting from day 5 as compared with baseline. In this group, the stiffness scores were reduced (*P* < 0.001) by 14.44, 19.15, and 30.19% on days 5, 14, and 30 of supplementation, respectively. These changes were also significantly compared with the placebo. Post-trial, the baseline WOMAC-B (Stiffness) scores in the CLBS and placebo groups were reduced by 10.93 and 7.35%, respectively. These improvements were not statistically significant as compared with the placebo and baseline (Table 3).

#### WOMAC physical function

The NXT15906F6 group exhibited significant improvements in physical function scores on days 14 and 30 of supplementation, as compared with placebo and baseline. NXT15906F6 supplementation resulted in 7.94, 16.38, and 27.95% reductions (*P* < 0.001) on days 5, 14, and 30 of supplementation, respectively, as compared with baseline. In contrast, at the end of the study, the CLBS group showed a 7.57% (*P* = 0.016) improvement in the WOMAC function score, while the change in score from the placebo was not significant (Table 3).

#### Visual analog scale

The ‘VAS Pain’ scores in the NXT15906F6 group were gradually and significantly reduced starting from day 5 through the end of the study. NXT15906F6 supplementation reduced the ‘VAS’ pain scores by 19.75, 27.33, and 33.10% on days 5, 14, and 30, respectively, as compared with the baseline. These improvements are also significant as compared with the placebo. In the CLBS-supplemented group, the pain score reductions were 2.71% (*P* = 0.607), 8.49% (*P* = 0.001), and 12.80% (*P* < 0.001) on days 5, 14, and 30, respectively, from baseline (Table 4).

**Table 4:** Musculoskeletal pain and function scores

Measures	Group	Evaluation	scores	change from baseline	*P* (vs. baseline)	95% CI vs. baseline	*P* (vs. placebo)	95% CI vs. placebo
Visual analog scale	Placebo	Baseline	52.21 ± 5.14	-	-	-	-	-
		Day 5	48.6 ± 5.05	−3.61 ± 2.63	0.006	1.45, 5.76	-	-
		Day 14	47.16 ± 5.13	−5.05 ± 3.53	<0.001	2.87, 7.22	-	-
		Day 30	45.5 ± 4.93	−6.71 ± 3.71	<0.001	4.57, 8.84	-	-
	NXT15906F6-250 mg	Baseline	50.79 ± 4.48	-	-	-	-	−0.59, 3.43
		Day 5	40.76 ± 4.11	−10.03 ± 4.13	<0.001	8.24, 11.81	<0.001	5.91, 9.76
		Day 14	36.91 ± 4.39	−13.88 ± 4.34	<0.001	12.04, 15.71	<0.001	8.25, 12.24
		Day 30	33.98 ± 4.65	−16.81 ± 4.52	<0.001	14.91, 18.70	<0.001	9.51, 13.52
	CLBS-1000 mg	Baseline	49 ± 5.34	-	-	-	-	1.00, 5.41
		Day 5	47.67 ± 4.45	−1.33 ± 4.87	0.607	−0.72, 3.38	0.596	−1.07, 2.93
		Day 14	44.84 ± 4.93	−4.16 ± 4.85	0.001	2.00, 6.31	0.064	0.20, 4.43
		Day 30	42.73 ± 5.73	−6.27 ± 4.82	<0.001	3.94, 8.59	0.032	0.52, 5.01
Lequesne’s Functional Index	Placebo	Baseline	12.49 ± 1.76	-	-	-	-	-
		Day 5	12.64 ± 1.45	0.15 ± 0.94	0.970	−0.53, 0.83	-	-
		Day 14	12.17 ± 1.45	−0.32 ± 1.34	0.773	−0.36, 1.00	-	-
		Day 30	12.08 ± 1.54	−0.41 ± 1.56	0.607	−0.29, 1.11	-	-
	NXT15906F6-250 mg	Baseline	13.30 ± 2.27	-	-	-	-	−0.04, 1.66
		Day 5	11.49 ± 1.81	−1.81 ±1.02	<0.001	0.95, 2.66	0.010	0.46, 1.83
		Day 14	10.88 ±1.86	−2.42 ± 1.29	<0.001	1.56, 3.27	0.005	0.58, 1.99
		Day 30	9.32 ± 1.68	−3.98 ±1.59	<0.001	3.15, 4.80	<0.001	2.08, 3.43
	CLBS-1000 mg	Baseline	12.39 ± 2.52	-	-	-	-	−0.81, 1.01
		Day 5	12.19 ± 2.44	−0.20 ± 0.68	0.985	−0.83, 1.23	0.989	−0.39, 1.29
		Day 14	12.00 ± 2.25	−0.39 ± 0.90	0.665	−0.61, 1.39	0.905	−0.62, 0.96
		Day 30	11.03 ± 2.39	−1.36 ± 1.23	0.012	0.33, 2.38	0.029	0.20, 1.89
Six minute walk test Absolute walk distance (m)	Placebo	Baseline	358.16 ± 17.39	-	-	-	-	-
		Day 5	362.23 ± 16.2	4.07 ± 2.97	0.650	−3.05, 11.9	-	-
		Day 14	365.07 ± 16.07	6.91 ± 5.94	0.200	−0.18, 14.00	-	-
		Day 30	372.93 ± 15.81	14.77 ± 8.84	<0.001	7.72, 21.81	-	-
	NXT15906F6-250 mg	Baseline	358.54 ± 15.37	-	-	-	-	−6.48, 7.24
		Day 5	391.39 ± 16.95	32.85 ± 16.09	<0.001	26.14, 39.55	<0.001	22.20, 36.11
		Day 14	413.91 ± 19.72	55.37 ± 20.55	<0.001	48.04, 62.69	<0.001	41.28, 56.39
		Day 30	475.00 ± 27.75	116.46 ± 25.87	<0.001	107.16, 125.75	<0.001	92.55, 111.58
	CLBS-1000 mg	Baseline	359.73 ± 14.45	-	-	-	-	−5.16, 8.30
		Day 5	362.30 ± 19.56	2.58 ± 18.11	0.931	−4.63, 9.77	1.000	−7.50, 7.65
		Day 14	371.69 ± 22.36	11.96 ± 21.29	0.029	4.07, 19.84	0.251	−1.60, 14.84
		Day 30	383.40 ± 23.6	23.67 ± 24.28	<0.001	15.47, 31.86	0.085	1.99, 18.95
Stair climb test (sec)	Placebo	Baseline	15.43 ± 0.68	-	-	-	-	-
		Day 5	15.6 ± 0.88	0.17 ± 0.68	0.953	−0.16, 0.50	-	-
		Day 14	15.13 ± 0.99	−0.30 ± 0.80	0.175	v0.05, 0.65	-	-
		Day 30	14.74 ± 1.05	−0.70 ± 0.86	0.117	0.31, 1.06	-	-
	NXT15906F6-250 mg	Baseline	15.60 ± 0.77		-	-	-	−0.13, 0.47
		Day 5	14.06 ± 1.04	−1.54 ±0.97	<0.001	1.16, 1.91	<0.001	1.13, 1.94
		Day 14	12.85 ± 1.24	−2.75 ± 1.17	<0.001	2.32, 3.17	<0.001	1.80, 2.75
		Day 30	10.56 ± 1.65	−5.04 ±1.63	<0.001	4.50, 5.57	<0.001	3.59, 4.76
	CLBS-1000 mg	Baseline	15.24 ± 0.90	-	-	-	-	−0.14, 0.52
		Day 5	15.22 ± 0.88	−0.02 ± 0.99	0.882	−0.35, 0.39	0.327	0.009, 0.75
		Day 14	14.67 ± 0.98	−0.57 ± 1.07	0.045	0.17, 0.96	0.856	0.04, 0.87
		Day 30	13.46 ± 1.13	−1.78 ± 1.28	<0.001	1.35, 2.20	<0.001	0.82, 1.73
30s Chair stand test no. of repetitions	Placebo	Baseline	15.93 ± 1.56	-	-	-	-	-
		Day 5	15.77 ± 1.76	−0.16 ± 0.98	0.291	−0.54, 0.86	-	-
		Day 14	16.43 ± 1.71	0.50 ± 0.93	0.010	−0.19, 1.19	-	-
		Day 30	16.75 ± 1.49	0.82 ± 1.10	0.001	0.17, 1.46	-	-
	NXT15906F6-250 mg	Baseline	16.02 ± 1.81	-	-	-	-	−0.61, 0.79
		Day 5	17.26 ± 1.87	1.24 ± 2.07	<0.001	0.47, 2.00	<0.001	0.72, 2.25
		Day 14	17.72 ± 2.07	1.70 ± 2.37	<0.001	0.89, 2.50	<0.006	0.49, 2.08
		Day 30	19.85 ± 2.59	3.83 ± 2.53	<0.001	2.90, 4.75	<0.001	2.20, 3.99
	CLBS-1000 mg	Baseline	17.02 ± 1.74	-	-	-	-	0.39, 1.78
		Day 5	17.18 ± 1.85	0.16 ± 1.90	0.587	−0.59, 0.91	0.001	0.64, 2.17
		Day 14	17.51 ± 2.00	0.49 ± 1.98	0.105	−0.29, 1.27	0.020	0.29, 1.86
		Day 30	18.04 ± 2.15	1.02 ± 2.26	0.001	0.20, 1.83	0.014	0.50, 2.07
Primary knee Flexion (°)	Placebo	Baseline	119.57 ± 1.52	-	-	-	-	-
		Day 5	119.18 ± 1.28	−0.39 ± 0.92	0.692	−0.20, 0.98	-	-
		Day 14	120.77 ± 1.65	1.20 ± 1.15	0.004	0.52, 1.87	-	-
		Day 30	122.14 ± 2.06	2.57 ± 2.16	<0.001	1.80, 3.33	-	-
	NXT15906F6-250 mg	Baseline	119.11 ± 2.03	-	-	-	-	−0.29, 1.21
		Day 5	121.91 ± 1.43	2.80 ± 2.23	<0.001	2.07, 3.52	<0.001	2.16, 3.29
		Day 14	123.28 ± 1.41	4.17 ± 2.48	<0.001	3.44, 4.89	<0.001	1.86, 3.15
		Day 30	125.48 ± 2.21	6.37 ± 2.98	<0.001	5.49, 7.24	<0.001	2.44, 4.23
	CLBS-1000 mg	Baseline	122.55 ± 1.7	-	-	-	-	2.30, 3.65
		Day 5	122.91 ± 2.01	0.36 ± 1.94	0.837	−0.41, 1.13	<0.001	3.01, 4.44
		Day 14	123.42 ± 2.21	0.87 ± 2.11	0.177	0.04, 1.69	<0.001	1.82, 3.47
		Day 30	123.73 ± 2.14	1.18 ± 2.29	0.051	0.37, 1.98	0.002	0.70, 2.47

Data presented as mean ± SD. Placebo (*n* = 44), NXT15906F6 (*n* = 46) and CLBS (*n* = 45). ‘Within the group’ and ‘between the groups’ comparisons were analyzed using paired *t*-test and ANCOVA, respectively. *P* < 0.05 indicate significance.

#### Lequesne functional index

The NXT15906F6 group demonstrated substantially (*P* < 0.001) decreased LFI scores by 13.61, 18.20, and 29.92% on days 5, 14, and 30 of supplementation, respectively, as compared with baseline. These changes are also significant as compared with the placebo. The CLBS group showed reductions in LFI scores by 1.61% (*P* = 0.985), 3.15% (*P* = 0.665), and 10.98% (*P* = 0.012) on days 5, 14, and 30 of supplementation, respectively, as compared with baseline (Table 4).

#### Six-Minute Walk Test

Absolute distance walked (AWD) in 6 min was evaluated at baseline and each follow-up visit of the study. The NXT15906F6 group showed increased walking distance starting from day 5 of the study and gradually improved till the end of the intervention. From baseline, the increases in AWD by the NXT15906F6-supplemented subjects were 9.16, 15.44, and 32.48% on days 5, 14, and 30 of supplementation, respectively. The improvements in AWD were significant (*P* < 0.001) as compared with the baseline and also with the placebo. In contrast, the CLBS-supplemented subjects showed only 6.58% (*P* < 0.001 vs. baseline; *P* = 0.085 vs. placebo) improvement in AWD at the end of the study (Table 4).

#### Stair Climb test

Time (in sec) taken to ascend and descend the flight of stairs was measured at baseline and each follow-up visit of the study. NXT15906F6 group showed significant reductions in time (in sec) taken to ascend and descend the flight of stairs on days 5, 14, and 30 as compared with baseline and the placebo. At the end of the study, NXT15906F6 and CLBS-supplemented participants exhibited 32.31% (*P* < 0.001) and 11.68% (*P* < 0.001) reductions in time taken for SCT, as compared with baseline. Interestingly, on day 5, the improvement in SCT by the NXT15906F6 participants was 9.78% (*P* < 0.001, vs. placebo; vs. baseline), while the CLBS group showed only a marginal improvement of 0.13% (*P* = 0.882 vs. baseline; *P* = 0.327 vs. placebo) (Table 4).

#### 30s chair stand test

The 30s-CST measures the number of times an individual can stand up from a chair in 30 sec with no assistance. On day 5, NXT15906F6-supplemented subjects demonstrated 7.74% improvement from baseline 30s-CST (*P* < 0.001 vs. baseline; vs. placebo). Post-trial, the improvements in the NXT15906F6 and CLBS-supplemented groups were 23.91 and 5.99%, respectively, from baseline. These changes were significant compared with the baseline and placebo (Table 4).

#### Primary Knee Flexion

Post-trial, the mean improvement in the primary knee flexion by the NXT15906F6-supplemented subjects was 5.35% (from baseline) (*P* < 0.001 vs. baseline; *P* < 0.001 vs. placebo); in contrast, the improvement in the CLBS-supplemented group was only 0.96% (*P* = 0.051 vs. baseline; *P* = 0.002 vs. placebo). Interestingly, the improvement in the NXT15906F6 group started from day 5 of the intervention; the ‘within’ and ‘between’ the groups’ comparison analyses revealed that the changes were significant (*P* < 0.001) (Table 4).

#### High-sensitivity C-reactive protein

**Fig. 3:**
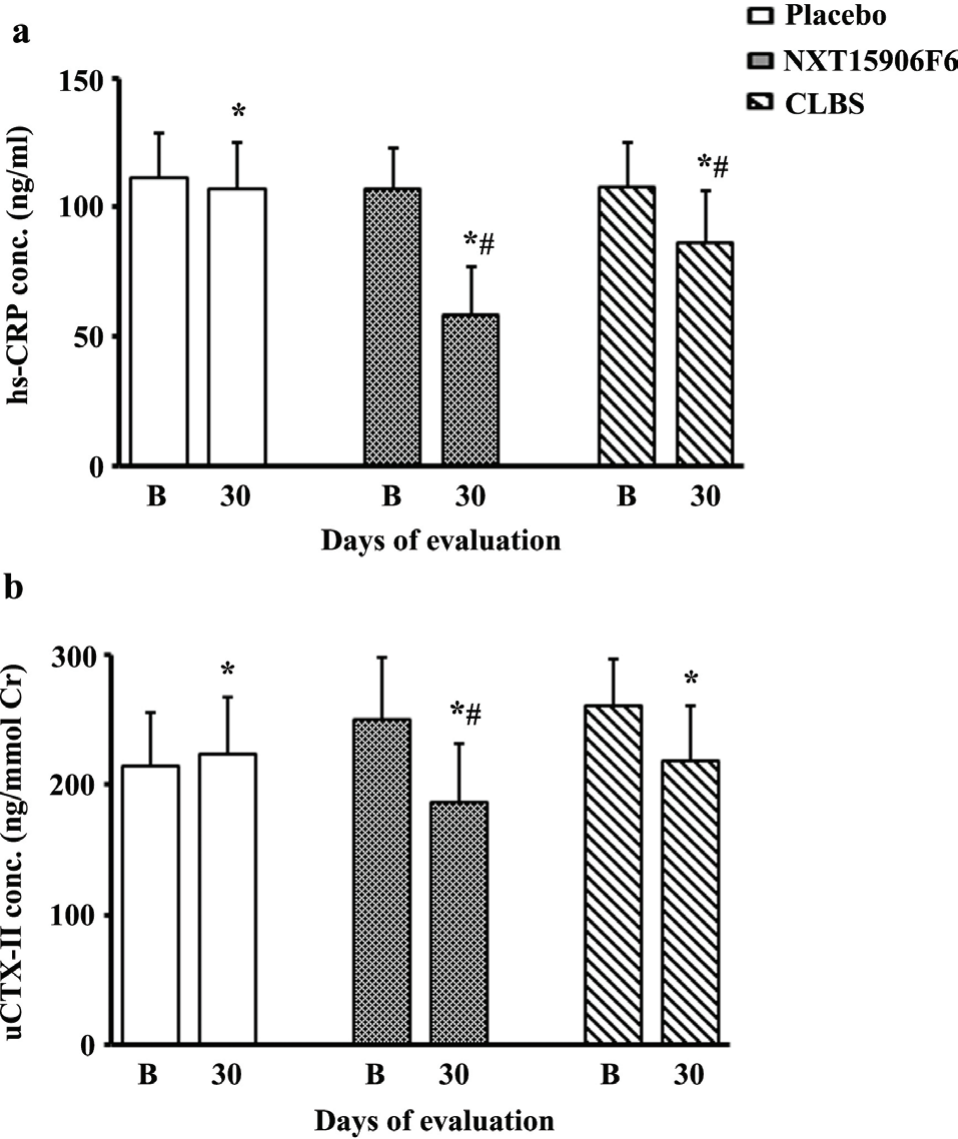
Supplementation of NXT15906F6 and CLBS reduced levels of serum hs-CRP (a), and urinary CTX-II (b) in the study participants. In each urine sample, CTX-II data were normalized with creatinine (Cr) concentration. Bars represent mean ± SD; Placebo (*n* = 44), NXT15906F6 (*n* = 46), and CLBS (*n* = 45), while * and ^#^ indicate significance (*P* < 0.05) in comparison analyses versus baseline (B) and versus placebo, respectively.

The NXT15906F6-supplemented group demonstrated a significant reduction in serum hs-CRP levels on day 30 as compared with the baseline. At the end of the study, the placebo, NXT15906F6-, and CLBS-supplemented groups exhibited 4.20, 45.14, and 20.07% reductions in the serum hs-CRP levels, respectively, from baseline. The changes in the serum hs-CRP levels in the active groups were significant (*P* < 0.001) as compared with the baseline and placebo (Fig. 3a).

#### Urinary C-telopeptide of type II collagen

NXT1506F6 and CLBS supplementation remarkably reduced the normalized uCTX-II levels from baseline. At the end of the trial, NXT15906F6 and CLBS-supplemented participants demonstrated 25.56% (*P* < 0.001) and 16.15% (*P* < 0.001) reductions in uCTX-II levels as compared with the baseline, respectively. Also, the change in the NXT15906F6 group was significant (*P* = 0.0003) as compared with the placebo. Post-trial, the placebo group showed a 4.40% (*P* = 0.0138) reduction in the uCTX-II level, as compared with the baseline (Fig. 3b).

## Safety evaluations

Evaluation of safety data includes all subjects who received at least one dose of the IP, that is, on the study’s (intention to treat) population. The vital signs, hematology, clinical biochemistry, and urinalysis results were within the normal ranges during the 30-day intervention (Supplementary Table 1).

## Adverse events, dropouts, and use of rescue medication

There were no adverse events reported by the participants during the study. No participant consumed any rescue medication. The number of dropped-out subjects from the study was 6, 4, and 5 from the Placebo, NXT15906F6, and CLBS groups, respectively. These participants were lost to follow-up; they were unavailable during the follow-up visits of the study.

## Discussion

The present 30-day intervention was conducted as a follow-up investigation in continuation to the previous studies (11, 12) to validate the clinical efficacy of NXT15906F6 in alleviating knee pain and improving musculoskeletal functions in OA subjects. Earlier, a 90-day randomized, double-blind, placebo-controlled study demonstrated that NXT15906F6 supplementation relieved knee pain and improved joint function following physical activity in non-arthritic male and female adults (12). Furthermore, in an independent clinical investigation, Kare et al. demonstrated that NXT15906F6 also significantly mitigated knee joint pain and improved musculoskeletal functions in the mild to moderate knee OA subjects (11). In addition to the clinical efficacy, the early onset of action of NXT15906F6 was also demonstrated by Kare et al. (11). The observations from the present study establish and validate the anti-OA efficacy and early onset of pain relief, improvements in musculoskeletal functions, and joint mobility in the male and female participants. Another objective of this study was to evaluate the clinical efficacy of a widely investigated combination of *C. longa* rhizome and *B. serrata* gum resin extracts (9). A meta-analysis performed on a series of preclinical and clinical studies supports that the blends of these two anti-inflammatory botanical extracts provide potential benefits in mitigating joint pain and improving musculoskeletal functions in OA subjects (9).

Knee pain and reduced joint function are the primary clinical symptoms of knee OA (23). Knee pain causes physical discomfort, deteriorates joint function, and reduces mobility and quality of life. In OA subjects, a perception of musculoskeletal pain is a major psycho-physiological limiting factor for restricting routine daily activities (24, 25). In this study, the significant improvements in WOMAC and VAS scores suggest that NXT15906F6 remarkably reduced OA joint pain in the participants. In addition, the NXT15906F6- supplemented participants also significantly improved the LFI score, increased the distance of travel in SMWT, reduced time in SCT, increased the number of repetitions in 30s-CST, and improved knee flexion. These improvements suggest that 30 days of supplementation of NXT15906F6 significantly improved knee joint movement, flexibility, and musculoskeletal functions, indicating increased lower body strength, power, and physical function in the NXT15906F6-supplemented volunteers. It is quite remarkable that the improvements in knee pain and musculoskeletal functional scores started as early as day 5 of supplementation in the NXT15906F6 participants. Interestingly, these observations are in agreement with the earlier findings, thus further establishing the validity of the clinical benefits of NXT15906F6 supplementation in the management of knee OA. It is also worth observing that although 30 days of supplementation of CLBS yielded significant improvements in the pain and functional scores, but failed to achieve an early efficacy, except for 30s-CST and primary knee flexion on day 5 of the intervention.

Osteoarthritis is an inflammatory and degenerative joint disorder of bone. In pathological conditions, elevated levels of TNFα, IL-1β and other selected inflammatory modulators are produced from inflamed synovial tissues, which further elevate production of pro-inflammatory cytokines, chemokines, and cartilage degrading enzymes, matrix metalloproteinases (MMPs) by autocrine and paracrine mechanisms (26–29). MMPs are powerful etiologic factors for the proteolytic degeneration of collagen in the cartilage tissue of joints in the onset and progression of OA (30). The data from the present investigation demonstrate that NXT15906F6 supplementation significantly reduced serum hs-CRP and urinary CTX-II levels. Although an increased serum hs-CRP level is poorly associated with OA grades but considered an indicator of systemic inflammation and pain (31). CTX-II is a cartilage degradation product; its elevated level in the urine is a hallmark indicative of OA progression (32). Collectively, the present observations indicate that NXT15906F6 reduced systemic inflammation and decreased proteolytic degradation of joint cartilage tissue in the participants. Thus, suggesting that the anti-inflammatory role of the botanical formulation in protecting the joint tissue to prevent OA progression. These findings further validate the earlier clinical data (11, 12). The polyphenolic proanthocyanidins of Tamarind seeds are anti-inflammatory and provide a defense against oxidative stress (7); curcuminoids are known to inhibit cyclooxygenase (COX-1 and COX-2) and reduce pro-inflammatory mediators, including TNF-α and IL-6 (33, 34). NXT15906F6 is a synergistic anti-inflammatory composition of tamarind seeds and *C. longa* extracts that reduces joint pain, inflammation and osteoarthritis symptoms associated with reduced levels of pro-inflammatory cytokines and MMPs in serum and cartilage tissue of monosodium iodoacetate (MIA)-induced Sprague Dawley rats (35).

Pain alleviation and improved joint functions are the significant benefits observed in the NXT15906F6-supplemented participants and these subjects experienced functional improvements from as early as 5 days of supplementation. This is the major strength of the present investigation. Also, an indication of reduced cartilage degeneration in the joint is a part of significant benefits of NXT15906F6 supplementation. However, one limitation of the study is that this is a short-duration study. A longer duration study in a larger population would help evaluate the beneficial efficacy of supplementation on knee point architecture in OA subjects.

Traditionally, Tamarind seeds (36) and turmeric rhizome (37) are used in food applications. Earlier, NXT15906F6 has been represented as a food-derived, safe, anti-inflammatory botanical composition (38). In addition to multiple clinical studies on NXT15906F6, a series of preclinical toxicity studies that included a 90-day sub-chronic repeated dose oral toxicity study and genotoxicity studies established the broad-spectrum safety of NXT15906F6. In the present investigation, the participants did not report any adverse events and their clinical chemistry parameters and vitals were within the normal ranges; these findings are in accordance with the earlier observations (11, 12).

## Conclusion

The present clinical investigation validates an early efficacy of a food-derived anti-inflammatory herbal composition NXT15906F6 (TamaFlex^TM^) in alleviating the clinical symptoms of knee OA in as early as day 5 of supplementation. NXT15906F6 reduces knee pain and improves the participants’ musculoskeletal function and joint mobility, resulting in improved physical performance and quality of life. NXT15906F6 supplementation also reduces the level of a cartilage erosion marker, indicating a possible role in mitigating joint tissue degradation. Importantly, this trial also reestablishes that NXT15906F6 is a tolerable and safe botanical composition for human consumption.

## Supplementary Material

Efficacy of a proprietary combination of *Tamarindus indica* seeds and *Curcuma longa* rhizome extracts in osteoarthritis: a clinical investigation
