# Supplementary material for: Efficacy of a proprietary combination of Tamarindus indica seeds and Curcuma longa rhizome extracts in osteoarthritis: a clinical investigation
Source: Food Nutr Res. 2023 Jun 20;67:10.29219/fnr.v67.9268. doi: 10.29219/fnr.v67.9268 (PMC10284097; doi:10.29219/fnr.v67.9268)
Supplement: Efficacy of a proprietary combination of Tamarindus indica seeds and Curcuma longa rhizome extracts in osteoarthritis: a clinical investigation [file FNR-67-9268-s001.doc]

**Supplementary Table 1: Safety parameters: hematology and clinical b**iochemistry

|  | **Parameter** | **Evaluations** | **Placebo** | **NXT15906F6** | **CLBS** |
| --- | --- | --- | --- | --- | --- |
| Hematology | Hemoglobin (g/dL) | Baseline | 13.61 ± 1.44 | 13.16 ± 1.82 | 12.97 ± 1.33 |
|  |  | Day 30 | 13.56 ± 1.22 | 13.36 ± 1.49 | 13.09 ± 1.21 |
|  | Platelet Count  (10^3^/cu. mm) | Baseline | 199.34 ± 72.55 | 198.24 ± 76.25 | 209.99 ± 80.99 |
|  |  | Day 30 | 208.15 ± 60.20 | 210.29 ± 72.10 | 203.44 ± 67.51 |
|  | ESR (mm/hr) | Baseline | 26.99 ± 22.79 | 27.64 ± 21.04 | 29.15 ± 22.82 |
|  |  | Day 30 | 22.14 ± 16.16 | 24.14 ± 19.63 | 25.30 ± 17.95 |
|  | RBC count  (10^6^/cu. mm) | Baseline | 4.78 ± 0.47 | 4.65 ± 0.52 | 4.61 ± 0.38 |
|  |  | Day 30 | 4.83 ± 0.46 | 4.77 ± 0.41 | 4.66 ± 0.49 |
|  | Total Leucocyte Count (10^3^/cu. mm) | Baseline | 8.19 ± 4.78 | 7.12 ± 1.94 | 7.36 ± 2.14 |
|  |  | Day 30 | 7.74 ± 2.05 | 7.57 ± 2.10 | 7.78 ± 2.40 |
|  | Hematocrit (%) | Baseline | 45.64 ± 5.11 | 44.69 ± 5.44 | 43.96 ± 4.37 |
|  |  | Day 30 | 44.33 ± 4.38 | 43.28 ± 4.68 | 43.36 ± 4.78 |
|  | Neutrophils (%) | Baseline | 60.77 ± 8.12 | 59.74 ± 7.19 | 61.39 ± 7.75 |
|  |  | Day 30 | 61.07 ± 6.61 | 60.84 ± 6.04 | 60.89 ± 5.32 |
|  | Lymphocytes (%) | Baseline | 31.50 ± 6.89 | 32.18 ± 6.96 | 30.64 ± 7.06 |
|  |  | Day 30 | 31.18 ± 5.50 | 30.78 ± 5.28 | 30.44 ± 5.28 |
|  | Eosinophil (%) | Baseline | 3.45 ± 2.02 | 3.77 ± 1.77 | 3.27 ± 1.54 |
|  |  | Day 30 | 3.43 ± 1.42 | 3.90 ± 1.88 | 3.94 ± 2.26 |
|  | Monocytes (%) | Baseline | 3.80 ± 2.10 | 3.82 ± 2.23 | 4.69 ± 2.77 |
|  |  | Day 30 | 3.79 ± 1.66 | 3.86 ± 1.91 | 4.10 ± 2.09 |
|  | Basophils (%) | Baseline | 0.48 ± 0.25 | 0.50 ± 0.27 | 0.48 ± 0.29 |
|  |  | Day 30 | 0.53 ± 0.24 | 0.61 ± 0.48 | 0.63 ± 0.48 |
| Serum Clinical Biochemistry | Fasting Blood Glucose (mg/dL) | Baseline | 95.53 ± 23.73 | 93.12 ± 13.82 | 92.15 ± 14.38 |
|  |  | Day 30 | 90.12 ± 9.72 | 92.32 ± 10.66 | 90.66 ± 9.58 |
|  | Serum Creatinine  (mg/ dL) | Baseline | 0.83 ± 0.20 | 0.82 ± 0.20 | 0.79 ± 0.20 |
|  |  | Day 30 | 0.77 ± 0.16 | 0.79 ± 0.18 | 0.76 ± 0.19 |
|  | BUN  (mg/ dL) | Baseline | 15.16 ± 5.19 | 15.00 ± 5.08 | 15.34 ± 5.32 |
|  |  | Day 30 | 16.29 ± 4.27 | 16.83 ± 4.70 | 16.03 ± 4.49 |
|  | BUA (mg/dL) | Baseline | 4.68 ± 1.35 | 4.78 ± 1.39 | 4.82 ± 1.12 |
|  |  | Day 30 | 5.06 ± 1.07 | 5.02 ± 1.12 | 4.72 ± 0.95 |
|  | Serum Sodium (meq/L) | Baseline | 139.28 ± 5.65 | 139.87 ± 4.12 | 140.12 ± 4.45 |
|  |  | Day 30 | 139.60 ± 2.76 | 140.32 ± 2.24 | 139.44 ± 3.35 |
|  | Serum Potassium (meq/L) | Baseline | 4.82 ± 0.59 | 4.79 ± 0.79 | 4.74 ± 0.53 |
|  |  | Day 30 | 4.85 ± 0.59 | 4.71 ± 0.72 | 4.63 ± 0.49 |
|  | ALT (IU/L) | Baseline | 27.13 ± 12.81 | 29.89 ± 14.25 | 28.07 ± 13.34 |
|  |  | Day 30 | 27.32 ± 9.09 | 27.14 ± 9.41 | 27.68 ± 10.65 |
|  | AST (IU/L) | Baseline | 28.29 ± 8.33 | 29.20 ± 9.16 | 26.93 ± 7.18 |
|  |  | Day 30 | 28.18 ± 9.04 | 28.69 ± 7.34 | 27.25 ± 7.58 |
|  | AP (IU/L) | Baseline | 101.02 ± 30.53 | 113.82 ± 31.14 | 108.82 ± 30.47 |
|  |  | Day 30 | 97.09 ± 32.51 | 101.00 ± 24.68 | 100.69 ± 26.44 |
|  | Bilirubin (mg/dL) | Baseline | 0.45 ± 0.20 | 0.44 ± 0.20 | 0.44 ± 0.24 |
|  |  | Day 30 | 0.45 ± 0.20 | 0.48 ± 0.21 | 0.45 ± 0.24 |
|  | Albumin (g/dL) | Baseline | 4.59 ± 0.36 | 4.57 ± 0.36 | 4.53 ± 0.33 |
|  |  | Day 30 | 4.48 ± 0.38 | 4.55 ± 0.41 | 4.50 ± 0.38 |
|  | Total Protein (g/dL) | Baseline | 7.62 ± 0.45 | 7.66 ± 0.40 | 7.57 ± 0.39 |
|  |  | Day 30 | 7.50 ± 0.34 | 7.55 ± 0.42 | 7.48 ± 0.32 |

Data presented as mean ± SD. At baseline, n=50; on day 30, Placebo (n=44), NXT15906F6 (n=46), and CLBS (n=45). BUA: Blood uric acid; BUN: Blood urea nitrogen; ALT: Alanine transaminase; AST: Aspartate transaminase; AP: Alkaline phosphatase.
